# Norethindrone Acetate in the Medical Management of Adenomyosis

**DOI:** 10.3390/ph5101120

**Published:** 2012-10-22

**Authors:** Ozgul Muneyyirci-Delale, Ashadeep Chandrareddy, Siddhi Mankame, Nanna Osei-Tutu, Hans von Gizycki

**Affiliations:** 1 Department of Obstetrics and Gynecology, SUNY Downstate Medical Center, 450 Clarkson Avenue, Brooklyn, NY 11203, USA; 2 Department of Obstetrics and Gynecology, Kings County Hospital Center, 451 Clarkson Avenue, Brooklyn, NY 11203, USA; 3 Scientific Computing Center, SUNY Downstate Medical Center, 450 Clarkson Avenue, Brooklyn, NY 11203, USA

**Keywords:** norethindrone acetate, adenomyosis, dysmenorrhea

## Abstract

The role of norethindrone acetate (NA) in the management of adenomyosis was evaluated with a retrospective chart review of 28 premenopausal women between 27–49 years of age presenting with moderate to severe pelvic pain and bleeding. Bleeding and dysmenorrhea scores were analyzed using paired T-tests. There was significant improvement of both dysmenorrhea and bleeding after treatment. Age showed no correlation with dysmenorrhea or bleeding. Low dose NA could be considered an effective, well-tolerated and inexpensive medical alternative to surgery for treating symptomatic adenomyosis. Large multicentric studies may help validate our findings.

## 1. Introduction

First described by Rokitansky in 1860 and then clearly defined by Von Recklinghausen in 1896, adenomyosis is a disorder characterized by the presence of islets within the myometrium that consist of both epithelial and stroma elements of endometrial tissues [1,2]. Characterized by symptoms of menorrhagia (50%), dysmenorrhea (30%) and metrorrhagia (20%), adenomyosis is a frequent and debilitating disease that is being encountered with increasing incidence in the infertile female population [3,4,5].

Adenomyosis is suggested to be an estrogen dependent condition [6]. The exact etiology is unknown but several theories have been proposed such as hereditary, hormonal influences, trauma, viral transmission and chronic postpartum endometritis. A significantly increased risk of adenomyosis has been found with prior uterine surgery [7].

Accurate determination of the prevalence of adenomyosis is difficult because, until recently, the diagnosis could only be made with certainty by microscopic examination of the hysterectomized uterus [8]. The systemic review and meta-analysis by Champaneria *et al*. in 2010, establishing the accuracy of transvaginal ultrasound and MRI in the preoperative diagnosis of adenomyosis. Since ultrasound is easily available in offices of most gynecologists, it seems to represent a real advance in the diagnosis of adenomyosis [9,10,11,12,13,14,15,16].

In those patients who are averse to surgery or those who wish to preserve their reproductive potential, newer, conservative medical and minor surgical procedures are increasingly being used in the treatment of adenomyosis. Norethindrone, a synthetic progestin, is the 17ά-ethinyl derivative of 19-nortestosterone and differs structurally from norethynodrel only in the position of the double bond in the A ring of the steroid. Norethindrone acetate (NA) is the acetic acid ester of norethindrone and is about twice as potent as norethindrone. It has been approved by the Food and Drug Administration. NA has been reported to be effective in the treatment of endometriosis [17]. Its long term efficacy has also been repeatedly demonstrated [18,19]. Okada *et al*. concluded that progestins inhibit estradiol-induced vascular endothelial growth factor and stromal cell-derived factor 1 in human endometrial stromal cells [20].

In lieu of this information and considering the pharmacological profile of NA, it may be presumed to be suitable for the management of adenomyosis. Thus, NA in the medical management of adenomyosis is a novel therapy. However, significant breakthrough bleeding is reported in patients of endometriosis being treated continuously with NA [21]. Due to differences in symptomotology and gland invasion of the two conditions, breakthrough bleeding may be deemed avoidable in the treatment of adenomyosis. Therefore ‘three-weeks-on and one-week-off’ regime has been employed in our treatment group.

## 2. Results

**Figure 1:**
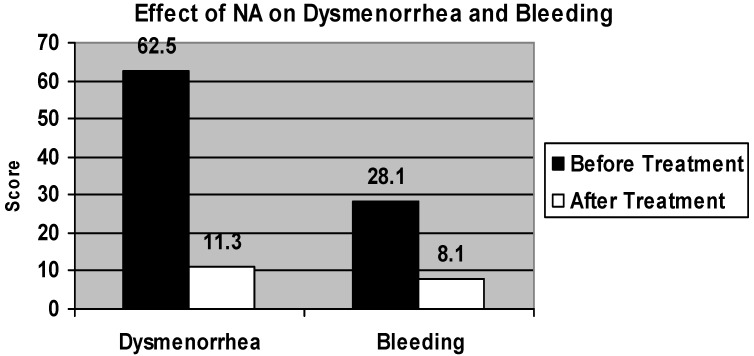
Dysmenorrhea score calculated by multiplying each day’s scores by total number of days of bleeding. Bleeding score calculated by adding the individual day’s scores.

Age of the women was in the range of 27–49 years with mean age being 40.67 ± 1.23. Mean BMI of the women was 31.38 ± 1.35. Patients showed maximum response to treatment at 3 months on NA and maintained throughout treatment. Both pain and bleeding showed significant decrease from pretreatment scores(*p* < 0.001). Dysmenorrhea scores before and after treatment were 62.5 ± 9.1 *vs.* 11.3 ± 3.1 respectively (*p* < 0.001). Bleeding scores before and after treatment were 28.1 ± 2.4 and 8.1 ± 8.5 respectively (*p* < 0.001) (Figure 1). Breakthrough bleeding was ascertained from patients at each visit. Six of the 28 patients (21.4%) reported breakthrough bleeding of a short duration. None of the patients reported breakthrough bleeding after 2 months.

## 3. Discussion

Adenomyosis is still a largely under diagnosed entity [22]. Transvaginal ultrasonography has been found to be as efficient as MRI for the diagnosis of adenomyosis in women without myoma, while MRI could be recommended for women with associated leiomyoma [23]. Besides, ultrasonography is widely available in most gynecological offices and is also a cheaper option. In our treatment group, ultrasound diagnosis was made using the characteristic findings of adenomyosis established by Levgur [15].

In a study conducted to compare the diagnostic potential of MRI and TVS in the diagnosis of adenomyosis, it was found that MRI had a higher specificity but their sensitivities were similar. The diagnostic accuracy of MRI and TVS was at an intermediate level but the diagnostic accuracy of the former improved by the exclusion of uteri of 400 mL. The combination of MRI and TVS produced the highest level of accuracy for exclusion of adenomyosis [24]. However, the diagnosis can only be made with certainty by histopathological analysis of the extirpated uterus [8].

The treatment of adenomyosis is often limited by the delay and difficulty with diagnosis. Although hysterectomy is considered to be the only procedure able to cure the disease definitively, various therapeutic options are currently available for symptomatic adenomyosis. 

Symptoms of adenomyosis may be alleviated by antiprogestins, sex hormones, danazol and GnRH analogs. Minor surgical procedures for therapy include endomyometrial ablation, laproscopic myometrial electrocoagulation and adenomyoma excision [25,26].

Focal and diffuse disease may be managed by laparoscopic electrocoagulation or myometrial excision with preservation of fertility but the risk of recurrence exists [26].

Uterine artery embolization presumably invokes infarction and necrosis but is more effective in women with accompanying predominant leiomyomata [27]. Hysterectomy is the ultimate solution for women with deep myometrial involvement or if future fertility is not desired [25].

Some preliminary studies have reported control of symptoms including dysmenorrhea and menorrhagia in women with adenomyosis using the 20 mcg/day levonorgestrel-releasing intrauterine system (LNG-IUS) [28]. Hormone suppression with GnRH agonists has also shown encouraging results [29,30]. An intrauterine contraceptive device (IUCD) loaded with an appropriate dose of danazol was found to be an effective treatment for adenomyosis in mice, in one study [31]. However, long term GnRH agonist therapy is not practical because of side effects associated with a hypoestrogenic state, the most serious being bone loss. Danazol therapy is limited by its androgenic side effects. These treatments are also associated with high recurrence rates [31].

Oral progestins have been reported to be effective in the treatment of endometriosis. The mode of action is still a matter of debate, but it may involve modulation of mitotic activity, local growth factors and growth factor receptors, as well as other paracrine mechanisms and anti-inflammatory reactions [32]. An advantage of this treatment modality is that, if symptoms recur after discontinuation of NA, the treatment can be repeated, unlike other minor surgical procedures and also other medical treatments known to cause bone loss with repeated use. 

Norethindrone, like other progestins, is known to cause breakthrough bleeding, and 21.4% of our patients reported such breakthrough bleeding, but never beyond two cycles of treatment. This was much less compared to the percentage of patients complaining of breakthrough bleeding who were on continuous NA treatment in previous studies (57.6% and 68%) [17,21]. Other less frequent symptoms, that weren’t reported by our patients, are changes in menstrual flow, amenorrhea, changes in cervical secretions, erosion, edema, weight gain or loss, cholestatic jaundice, allergic rash with or without pruritus, melasma or chloasma and mental depression. In this treatment group, the ‘three weeks on, one week off’ regime was adopted to minimize the previously reported side effect of breakthrough bleeding that we observe with treatment of endometriosis. This regime causes incomplete suppression of the hypothalamic-pituitary-ovarian axis, perhaps causing less of a hypoestrogenic effect and less breakthrough bleeding. This hypothesis could be further explored in future studies comparing continuous *vs.* interrupted treatment.

Our retrospective chart review indicates that marked relief from adenomyosis associated menorrhagia and pain can be obtained with the use of NA. Despite shortcomings such as the small sample size, relatively short follow-up and no objective measure used for uterine bleeding and no other method used to quantify dysmenorrhea and no follow-up after discontinuation of treatment. The results show that NA is effective in relieving dysmenorrhea and menorrhagia. 

As of now, no documentation exists about the histological, sonographic and MRI appearance of adenomyosis before and after treatment with norethindrone acetate. Also, limitations of this review need to be systematically addressed in future studies by conducting a long term follow up after discontinuation of NA treatment of adenomyosis, hormonal, sonographic or MRI evaluations of patients while on treatment and studying the effect of the treatment on the Hypothalamic-Pituitary-Ovarian Axis. Large multicenter studies comparing NA to other progestins or levonorgestrel releasing intrauterine system may help validate our findings.

## 4. Material and Methods

This is a retrospective chart review at a university-based hospital. Institutional review board approval was obtained prior to its commencement. The review included 28 premenopausal women who presented with moderate to severe dysmenorrhea and menorrhagia. They were diagnosed with adenomyosis and treated with NA. All 28 patients were symptomatic, with complaints of moderate to severe dysmenorrhea and menorrhagia of at least 6 months’ duration. Age of the women was in the range of 27–49 years.

Adenomyosis was diagnosed based on the clinical presentation (menorrhagia, dysmenorrhea, diffusely enlarged uterus) and transvaginal ultrasound. According to Levgur, the characteristic findings on ultrasound, for the diagnosis of adenomyosis, are divided into those of the uterine body, myometrium and endometrium. Some of the changes involving the myometrium include: focal honeycomb scattered, irregular, cystic, anechoic lacunae, mottled texture, asymmetric thickening of anterior or posterior uterine wall [15].

Endometrial biopsy was done in all women aged more than 35 years. Magnetic Resonance Imaging (MRI) was used to confirm adenomyosis according to the criteria of Togashi *et al.* [33]. The patients who were selected for treatment included those who refused surgery, preferring a medical therapy and who did not have submucosal myomas, adnexal disease, endometriosis and endometrial pathology such as polyp, hyperplasia and endometrial cancer.

Prior to commencing treatment, all the women were counseled about other treatment options. They were informed that medical treatments, previously used for adenomyosis, generally induce only temporary relief and that only surgery is definitively curative. They were also informed that since endometriosis and adenomyosis are considered one pathophysiological entity, we were using NA to treat adenomyosis. Also, these women were explained the proposed mechanism of the effect of NA on their symptoms and the possibility of treatment failure, recurrence, and the eventual need for hysterectomy.

A low dose (5 mg) of NA was chosen for treatment and initiated at the beginning of the menstrual cycle. NA was taken orally as a three-weeks-on and one-week-off regime. Since maximum response was obtained at 3 months in our previous studies, patients were seen every 3 months during treatment [17,18].

As a part of their routine evaluation, we documented the presence and severity of dysmenorrhea by grading it on a visual analogue scale of 0 to 10 (0: no pain, 10: worst possible pain; 1–3: mild, 4–6: moderate, 7–10: severe). The dysmenorrhea score was developed by multiplying the grade by the number of days that the patient pain (0–10, multiplied by the number of days). Bleeding scores (O. Muneyyirci-Delale modified menstrual calendar, added scale 4: heavy with clots), documented on a scale of 0–4, were used to estimate the amount of bleeding (scale of 0–4: heavy with clots = 4, heavy = 3, normal = 2, light = 1, no bleeding = 0, all the individual days’ scores were added). Patients with scale of 3 and 4 were treated with NA.

Response to hormonal treatment was ascertained verbally from patient for bleeding and pain scores obtained during the follow-up visits. Bleeding and pain scores, before and after treatment, were compared. Student’s T-test was used for statistical analysis.

## 5. Conclusions

We suggest that NA offers promise as an effective and well tolerated drug in the management of symptomatic adenomyosis. We propose to undertake a double-blinded randomized controlled clinical trial to evaluate the role of NA in the management of symptomatic adenomyosis to provide answers to some of the questions raised by this retrospective chart review. NA may be a much cheaper alternative to other treatment options for adenomyosis, with fewer and milder side effects.
